# Low SIRT3 Expression Correlates with Poor Differentiation and Unfavorable Prognosis in Primary Hepatocellular Carcinoma

**DOI:** 10.1371/journal.pone.0051703

**Published:** 2012-12-14

**Authors:** Chris Zhiyi Zhang, Lili Liu, Muyan Cai, Yinghua Pan, Jia Fu, Yun Cao, Jingping Yun

**Affiliations:** 1 State Key Laboratory of Oncology in South China, Sun Yat-sen University Cancer Center, Guangzhou, China; 2 Department of Pathology, Sun Yat-sen University Cancer Center, Guangzhou, China; 3 Department of Rheumatology and Immunology, The Third Affiliated Hospital of Sun Yat-sen University, Guangzhou, China; University of Hong Kong, Hong Kong

## Abstract

SIRT3, a mitochondrial sirtuin belonging to nicotinamide adenine nucleotide (NAD) dependent deacetylases, is implicated in metabolism, longevity and carcinogenesis. SIRT3 expression and its significance in hepatocellular carcinoma (HCC) remain largely unclear. In this study, we demonstrated that SIRT3 expression in HCC tissue was much lower than that in paracarcinoma tissue, at both mRNA and protein levels. The cutoff value for low SIRT3 expression in HCC was defined according to receiver operating characteristic curve (ROC) analysis. As disclosed by immunohistochemistry (IHC) results, low SIRT3 expression was present in 67.3% (167/248) of HCC cases. Furthermore, low expression of SIRT3 was significantly correlated to differentiation (*P* = 0.013), clinical stage (*P* = 0.005), serum AFP level (*P*<0.01), tumor multiplicity (*P* = 0.026) and relapse (*P* = 0.028). Moreover, Kaplan-Meier analysis indicated that low SIRT3 expression associated with unfavorable overall survival (*P*<0.01) and recurrence-free survival (*P* = 0.004). The prognostic impact of SIRT3 was further confirmed by stratified survival analysis. Importantly, multivariate analysis revealed that low SIRT3 expression was an independent poor prognostic marker for overall survival (Hazard Ratio (HR) 0.555, 95% confidence interval (95% CI) 0.344–0.897, *P* = 0.016). Collectively, we conclude that SIRT3 is decreased in HCC and is a novel unfavorable marker for prognosis of patients with this fatal disease.

## Introduction

Hepatocellular carcinoma (HCC) is the fifth most prevalent cancer, and ranks third as a cause of cancer death worldwide [1]. Incidence has been increasing in economically developed regions, including Japan, Western Europe, and the United States in recent decades [2], [3]. Although new strategies have been applied for HCC treatment, efficacies are still beyond satisfactory [4]. In view of that the poor prognosis of HCC, with a median survival time of 4 months [1], and that the accuracy and reproducibility of markers current used in clinic to predict survival after surgical resection remain either unsatisfactory or unclear [5], it is of immense importance to develop new/novel prognostic factors.

Sirtuin family, the human homologues of the Sir2 gene in yeast [6], function either as nicotinamide adenine dinucleotide (NAD)^+^-dependent deacetylases or as ADP-ribosyl transferases [7], [8]. Sirtuins have been demonstrated to play important roles in many physiological and pathophysiological conditions, such as cell survival, metabolism, aging, longevity, and carcinogenesis [9], [10], [11]. SIRT3, which is genomically expressed and highly conserved through evolution, preferentially localized to mitochondria with its N-terminal 25 amino acid residues [12]. It is a mitochondrial matrix protein with high amount in mouse kidney, heart, and liver tissues [13]. The first identified substrate of SIRT3 was Acetyl-CoA Synthetase 2 (AceCS2) [14]. Although plenty of literatures supported SIRT3 was involved in mitochondrial energy production and substrate oxidation [15], expression of SIRT3 in cancer has been controversial. For example, Ashraf et al. reported that SIRT3 was markedly increased in lymph node-positive breast cancer biopsies, compared to the normal tissues [16]. However, in another study, significant decrease of SIRT3 was observed in 992 human breast cancer samples [17]. SIRT3 was demonstrated to increase in oral squamous cell carcinoma (OSCC) cell lines and human OSCC tissue samples [18]. Recently, SIRT3 was shown to downregulated in 4 paired HCC tissues, compared to the adjacent liver tissues [19]. Based on the discrepancy in the current literatures, to clearly investigate the expression of SIRT3 and clinical significance in different types of cancer is of particular interests in developing SIRT3 to a promising therapeutic target in cancer treatment.

In the present study, the expression of SIRT3 and its clinical significance in HCC were investigated. We examined SIRT3 expression in HCC cell lines and human tissue samples, evaluated the association of SIRT3 expression and clinicopathological variables, and assessed the role of SIRT3 in HCC prognosis. Our data showed a noticeable decrease of SIRT3 in HCC and significant correlations of SIRT3 expression with clinical parameters and overall survival of HCC patients.

## Materials and Methods

### Cell Culture

Non-tumorigenic immortalized liver cell line (MiHA) was kindly provided by XY Guan from The University of Hong Kong and maintained in Dulbecco’s modified Eagle’s medium (DMEM) (Gibco, Gaithersburg, MD, USA). PLC/PRF/5 and SK-hep-1 cell lines were purchased from American Type Culture Collection (ATCC, Manassas, VA), and cultured in DMEM containing 10% fetal bovine serum (FBS), 100 mg/ml penicillin, and 100 mg/ml streptomycin. SMMC-7721, Bel-7404, Bel-7402, Huh7, HepG2 and QSG-7703 cell lines, obtained from the Type Culture Collection Cell Bank, Chinese Academy of Science Committee (Shanghai, China), were maintained in Roswell Park Memorial Institute (RPMI) 1640 with 10% of fetal bovine serum (FBS), 100 mg/ml of penicillin, and 100 mg/ml of streptomycin. All cells were incubated in a humidified atmosphere of 5% CO_2_ and 95% air at 37°C.

### Patients and Tissue Specimens

In this study, all primary HCC specimens along with complete clinical and pathological data were collected from 248 HCC patients who underwent surgical resection at Sun Yat-Sen University Cancer Center (SYSUCC), Guangzhou, China, between Jan 1997 and Dec 2007. The cohort consists of 220 (88.7%) man and 28 (11.3%) female. The mean age is 47.8, ranged from 14.0 to 78.0. Postsurgical survival data were available for all 248 patients. Average follow-up time was 32.1 months (median, 27.0 months; range, 1.0 to 86.0 months). Another 16 paired fresh resection HCC tissues and the corresponding adjacent liver tissues were collected for quantitative real-time PCR and western blot analysis. None of the patients had received adjuvant therapies before surgery. Tumor stage was defined according to tumor-node-metastasis (TNM) classification of the American Joint Committee on International Union against Cancer. Tumor differentiation was assessed according to Edmonson and Steiner grading system. The use of tissues for this study has been approved by the Institute Research Medical Ethics Committee of SYSUCC. No informed consent (written or verbal) was obtained for use of retrospective tissue samples from the patients within this study, most of whom were deceased, since this was not deemed necessary by the Ethics Committee, who waived the need for consent. All samples were anonymous.

### Tissue Microarray (TMA) Construction

TMA containing of 248 HCC and adjacent paracarcinoma liver tissues were constructed in light of the previously described method [20]. Briefly, all specimens were fixed in 4% formalin and embedded in paraffin. The corresponding histological HE-stained sections were reviewed by a senior pathologist to mark out representative areas. Using a tissue arraying instrument (Beecher Instruments, Sliver Spring, MD), each tissue core with a diameter of 0.6 mm was punched from the marked areas and re-embedded.

### Immunohistochemistry (IHC)

Formalin-fixed and paraffin-embedded HCC sections with a thickness of 4 µm were dewaxed in xylene and graded alcohols, hydrated, and washed in phosphatebuffered saline (PBS). After pretreatment in a microwave oven, endogenous peroxidase was inhibited by 3% hydrogen peroxide in methanol for 20 min, followed by avidin-biotin blocking using a biotin-blocking kit (DAKO, Germany). Slides were then incubated with SIRT3 antibody, overnight in a moist chamber at 4°C, washed in PBS, and incubated with biotinylated goat anti-rabbit/mouse antibodies. Slides were developed with the Dako Liquid 3, ’3-diaminobenzidine tetrahydrochloride (DAB)+Substrate Chromogen System and counterstained with hematoxylin.

### Quantitative Real-time PCR (qRT-PCR)

Total RNA was extracted from paired HCC samples, following the Trizol reagent (BIOO Scientific Co., USA) manufacturer’s instruction. mRNA was reversed to cDNA by M-MLV Reverse Transcriptase (Promega Inc., USA). Levels of SIRT3 and β-actin were measured by SYBR green-based real-time PCR using the Stratagene Mx3000P Real-Time PCR system. Primers were designed as follows: SIRT3, forward: 5′-GCATTCCAGACTTCAGATCGC-3′ and reverse: 5′-GTGGCAGAGGC AAAGGTTCC-3′; β-actin, forward: 5′-TGGCACCCAGCACAATGAA-3′ and reverse: 5′-CTAAGTCATAGTCCGCCTAGAAGC A-3′. Conditions were set as follows: one cycle of 95°C for 10 min, followed by 40 amplification cycles at 95°C for 10 s, annealing at 58°C for 20 s and elongation at 72°C for 15 s. Using the comparative threshold cycle (2^−ΔCt^) method, the relative expression of SIRT3 in HCC were normalized to the endogenous β-actin.

### Western Blot

Cell or tissue lysates were boiled with 6X sodium dodecyl sulfate (SDS) loading buffer and then fractionated by SDS-PAGE. The proteins were transferred to PVDF membrane which was then incubated with a primary specific antibody for SIRT3 in 5% of non-fat milk, followed by a horse radish peroxidase (HRP)-conjugated anti-rabbit second antibody. ECL detection reagent (Amersham Life Science, Piscataway, NJ, USA) was used to demonstrate the results.

### IHC Evaluation

Semi-quantitative IHC detection was used to determine the SIRT3 protein levels. A brown particle in nuclei was considered as positive labeling. Immunostain was scored using a 4-point scale (0–4) system according to the intensity of staining and the percentage of positive cells. IHC evaluation was performed according to the method described before [21]. For each case, 1000 cells were randomly selected and scored. HCC sections were observed under light microscopy and the staining intensities scores were independently assessed by 2 pathologists (Dr. JP Yun and Dr. MF Zhang).

### Selection of Cutoff Score

Receiver operating characteristic (ROC) curve analysis was employed to determine the cutoff score for tumor with low SIRT3 expression by using the 0,1-criterion. In immunohistochemical evaluation, the score with the shortest distance from the curve to the point with both maximum sensitivity and specificity, i.e., the point (0.0, 1.0), was selected as the cutoff score leading to the largest number of tumors correctly classified as having or not having the clinical outcome [22], [23]. At SIRT3 score, the sensitivity and specificity for each outcome under study was plotted, thus generating various ROC curves. The count was selected as the cutoff value, which was closest to the point with both maximum sensitivity and specificity. Cases defined as high SIRT3 expression were those with the scores below or equal to the cutoff value, while low SIRT3 expression represented those with scores above the value. In order to perform ROC curve analysis, clinicopathological features were dichotomized: tumor multiplicity (single vs multiple), tumor size (<5 cm vs ≥5 cm), AFP level (<20 ng/ml vs ≥20 ng/ml), tumor differentiation (well-moderate vs poor-undifferentiated), stage (I+II vs III+IV), vascular invasion (yes vs no), relapse (yes vs no) and survival status (dead vs alive).

### Statistical Analysis

Statistical analyses were performed using the SPSS 16.0 software (SPSS,Chicago, IL, USA). ROC curve analysis was applied to determine the cutoff value for high expression of SIRT3 by the 0,1-criterion, and the areas under curve (AUC) were calculated. Mann-Whitney U test was used for comparison between groups. Wilcoxon matched paired test was used to determine the significance of SIRT3 expression in fresh HCC and normal liver tissues. χ^2^ test was performed to analyze the correlation between SIRT3 expression and clinicopathological parameters. Kaplan-Meier method (the log-rank test) was utilized for survival analysis and univariate analysis. Independent analyses were performed according to the selected population: overall population and different morphological and pathological subgroups. Cox proportional hazards regression model was used to identify the independent prognostic factors. *P*<0.05 (two-tailed) was considered statistically significant.

## Results

### SIRT3 Expression in HCC Cell Lines and Tissues by qRT-PCR and Western Blot

**Figure 1:**
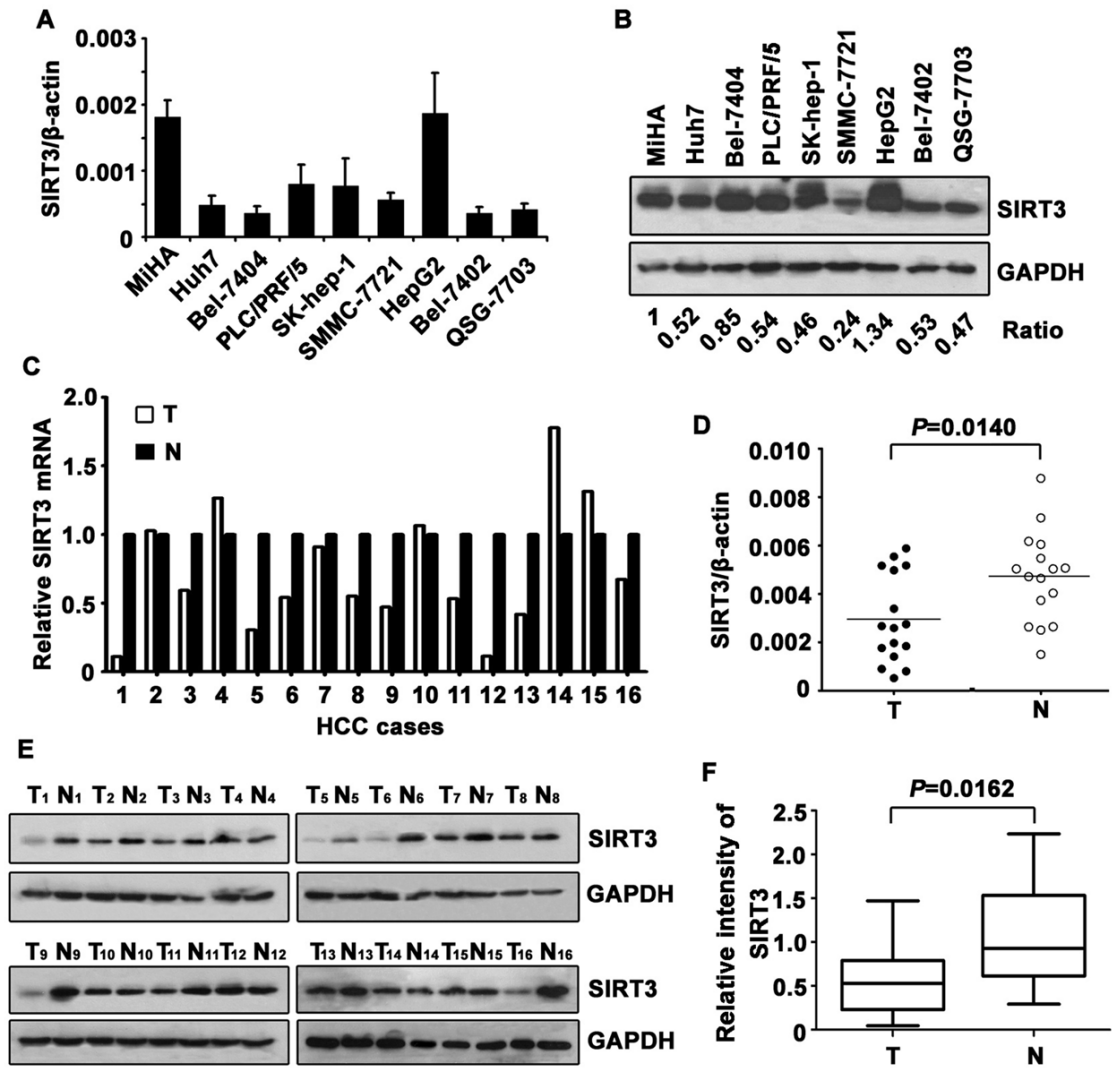
Expression of SIRT3 in HCC cell lines and tissue samples. A. mRNA level of SIRT3 in immobilized liver cell line (MiHA) and HCC cell lines was determined by qRT-PCR. Three independent experiments were performed. Data are mean ± SD. **B.** Representative pattern of SIRT3 protein expressed in cell lines was shown. The ratio of SIRT3/GAPDH was indicated as well. **C.** mRNA level of SIRT3 in HCC and corresponding adjacent liver tissue was determined in 16 patients. Relative SIRT3 mRNA in HCC tissues was presented. **D.** Wilcoxon matched paired test revealed the significant alteration of SIRT3 mRNA in tissue samples. **E.** Expression of SIRT3 protein in 16 paired HCC and adjacent normal liver tissues were examined by western blot. **F.** Relative intensity of PLK4 normalized to GAPDH was calculated.

To determine the expression pattern of SIRT3 in HCC, we firstly examine its level in immortalized liver cell lines and HCC cell lines. Results showed that SIRT3 was noticeably decreased in HCC cell lines, compared to the MiHA cell line, at both mRNA (Fig. 1A) and protein (Fig. 1B) levels.

Decrease of SIRT3 was further investigated in 16 paired fresh tissue samples. SIRT3 mRNA was significantly downregulated in 10 out of 16 cases in HCC tissues, compared to the corresponding adjacent liver tissues (Fig. 1C). Consistently, expression of SIRT3 protein was noticeably lower in HCC than that in paracarcinoma tissue in 68.8% of cases (Fig. 1E). The differential expression of SIRT3 in tumor and adjacent nontumor tissues appeared statistically significant (Fig. 1D and 1F).

### Definition of Cutoff Score for Low SIRT3 Expression in HCC

**Figure 2:**
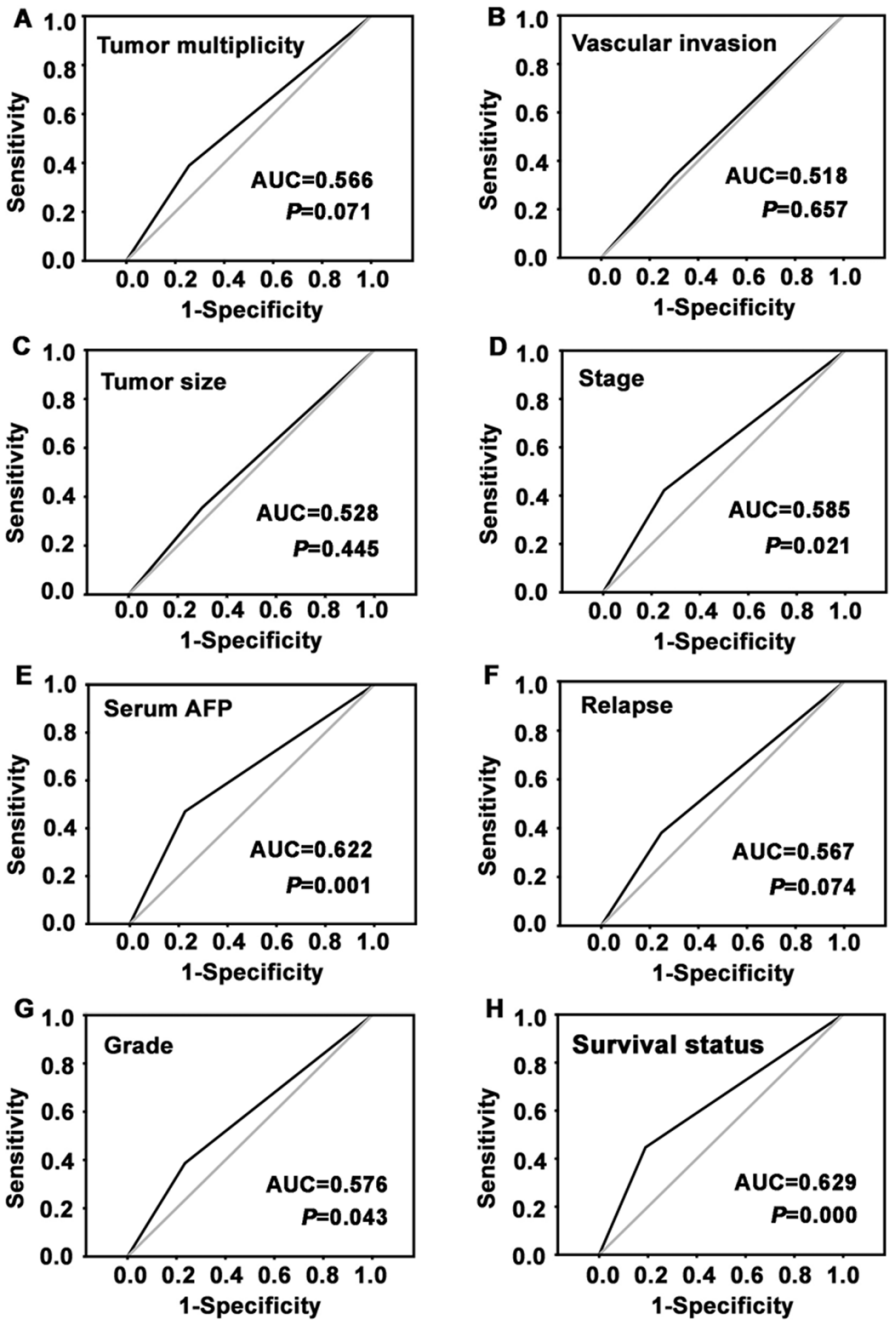
Determination the cutoff value of low SIRT3 expression in primary HCC tissues by receiver operating characteristic (ROC) curves. For each parameter of HCC patients, including tumor multiplicity, tumor size, serum AFP, pathological grade, clinical stage, vascular invasion, relapse and survival status, the sensitivity and 1-specificity were plotted. The areas under curve (AUC) and the *P* value were indicated.

In order to better assess the expression of SIRT3 in HCC, we employed ROC curve analysis to define an optimal cutoff value for low SIRT3 expression, based on the results of IHC evaluation. Results showed that ROC curve analysis for survival status has the shortest distance from the curve to the point (0.0, 1.0) (Fig. 2). Thus, we selected the cutoff value for survival status. Tumors with scores below the obtained cutoff value were considered to be with low SIRT3 expression, leading to the greatest number of tumors correctly classified as having (i.e., case group) or not having (i.e., control group) the positive clinical outcome. As a result, the cutoff score for low SIRT3 expression is 2.50, which indicated that tumor were defined as one with low SIRT3 expression when the IHC score was not higher than 2.50.

### Association between SIRT3 Expression and Clinicopathological Variables

**Figure 3:**
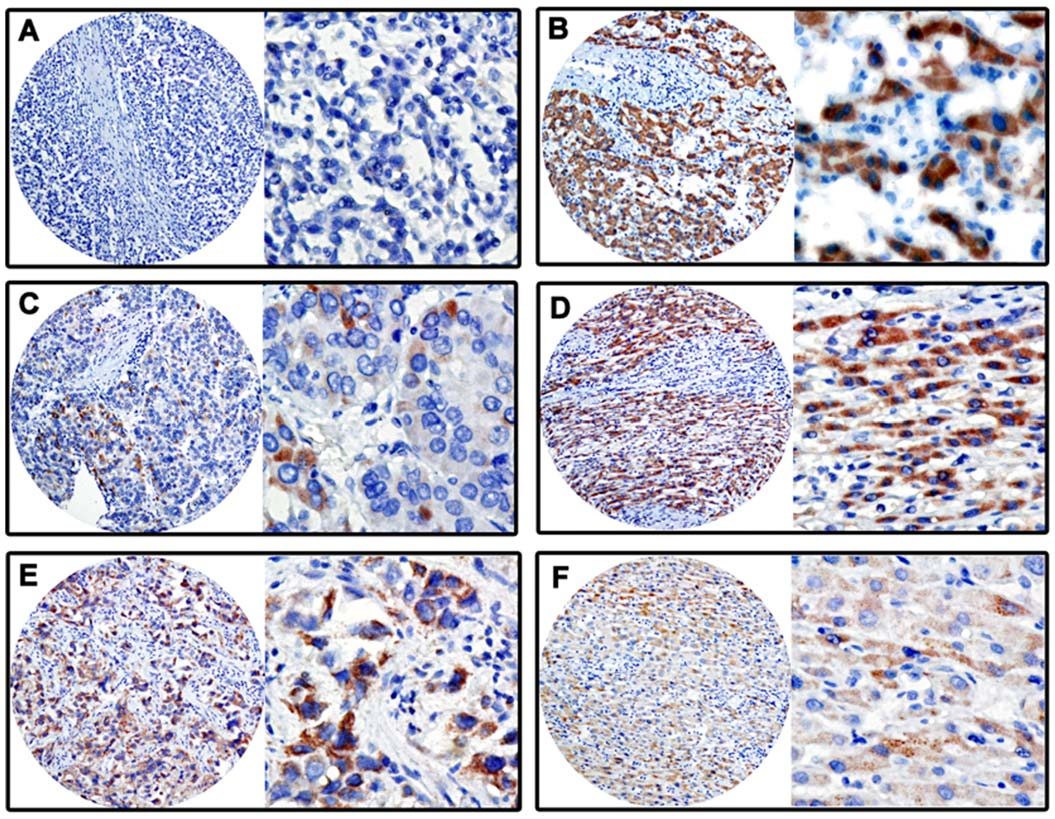
Protein expression patterns of SIRT3 in HCC tissues by IHC. The immunoreactivities were primarily observed in cytoplasm within tumor and normal liver cells. The micrographs showed negative (**A**), low (**C**), and high (**E**) expression of SIRT3 in HCC tissues. The relevant expressions of SIRT3 in corresponding adjacent normal liver tissues of cases in **A**, **C** and **E** were shown in **B**, **D** and **F**, respectively. (Left panel: magnification ×100; Right panel: magnification ×400.)

Since SIRT3 was remarkably downregulated in HCC cell lines and cancer tissues, we next further examined its expression in 248 paraffin-embedded HCC tissues. According to the results of TMA-based IHC, SIRT3 was mainly presented in cytoplasm (Fig. 3A–F). Out of more than 65% of cases, SIRT3 expressions in HCC were lower than those in corresponding adjacent nontumor tissues. Furthermore, based on the definition of cutoff score, low SIRT3 expression was observed in 67.3% (167/248) of HCC cases.

To determine the clinical significance of SIRT3 expression in HCC, relationship between SIRT3 expression and clinicopathological features was analyzed. Significant correlations were found between SIRT3 expression and variables including differentiation (*P* = 0.013), clinical stage (*P* = 0.005), serum AFP level (*P*<0.01), tumor multiplicity (*P* = 0.026) and relapse (*P* = 0.028). HCC patients with low SIRT3 expression had a higher tendency to be with poor differentiation, advanced stage, high level of serum AFP and multiple tumor numbers. There were no statistical connections between SIRT3 expression and the other clinicopathological parameters, such as age, gender, HBsAg, cirrhosis, tumor size and vascular invasion (*P*>0.05, Table 1).

**Table 1:** Correlation between the clinicopathologic variables and SIRT3 expression in HCC.

Variable	SIRT3 protein				
	All cases	Low expression	High expression	χ^2^	*P* value^a^
Age (years)^b^				0.021	0.884
<47.8	130	87 (66.9%)	43 (33.1%)		
≥47.8	118	80 (67.8%)	38 (32.8%)		
Gender				0.842	0.359
Female	28	21 (75.0%)	7 (25.0%)		
Male	220	146 (66.4%)	74 (33.6%)		
HBsAg				0.096	0.756
Positive	215	144 (67.0%)	71 (33.0%)		
Negative	33	23 (69.7%)	10 (30.3%)		
AFP (ng/ml)				16.521	**0.000**
<20	102	54 (52.9%)	48 (47.1%)		
≥20	146	113 (77.4%)	33 (22.6%)		
Cirrhosis				0.004	0.949
Yes	180	121 (67.2%)	59 (32.8%)		
No	68	46 (67.6%)	22 (32.4%)		
Tumor size (cm)				0.889	0.346
<5	121	78 (64.5%)	43 (35.5%)		
≥5	127	88 (69.3%)	39 (30.7%)		
Tumor multiplicity				4.936	**0.026**
Single	131	80 (61.1%)	51 (38.9%)		
Multiple	117	87 (74.4%)	30 (25.6%)		
Differentiation				6.224	**0.013**
Well-Moderate	150	92 (61.3%)	58 (38.7%)		
Poor-Undifferentiation	98	75 (76.5%)	23 (23.5%)		
Stage				8.048	**0.005**
I–II	109	63 (57.8%)	46 (42.2%)		
III–IV	139	104 (74.8%)	35 (25.2%)		
Vascular invasion				0.300	0.584
Yes	73	51 (69.9%)	22 (30.1%)		
No	175	116 (66.3%)	59 (33.7%)		
Relapse				4.864	**0.028**
Yes	101	76 (75.2%)	25 (24.8%)		
No	147	91 (61.9%)	56 (38.1%)		

a Chi-square test;

b Mean age;

AFP, alpha-fetoprotein; HBsAg, hepatitis B surface antigen.

### Interrelation of SIRT3 Expression and HCC Differentiation

**Figure 4:**
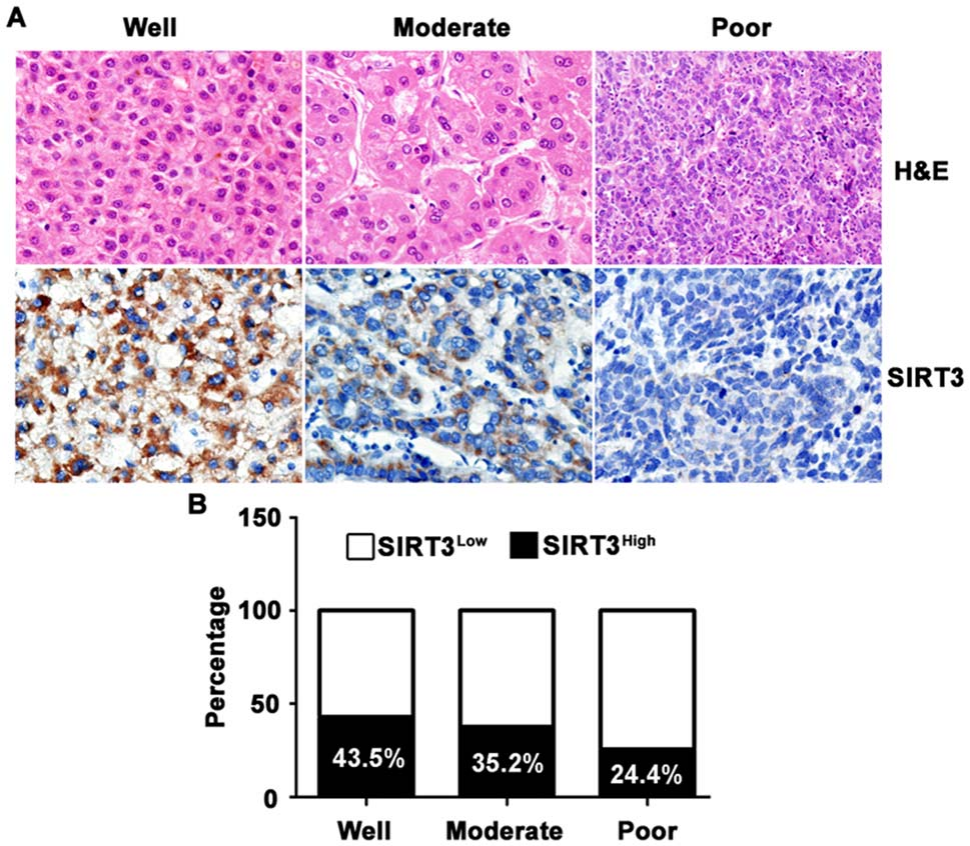
Reverse association of SIRT3 expression in HCC tissue with tumor differentiation. **A.** Representative micrographic images were shown to present the correlation between SIRT3 expression in HCC and differentiation. **B.** Percentages of high SIRT3 expressions in differentiated HCC were indicated by histogram.

As indicated in Table 1, expression of SIRT3 was related to HCC differentiation. We next further confirmed the reverse connection of SIRT3 expression in HCC and tumor differentiation. Another 30 HCC cases (10 cases in each group of well, moderate and poor differentiation) diagnosed from Mar 2011 to Oct 2011 were collected to determine the SIRT3 expression patterns. Results showed that SIRT3 expression in noncancerous tissue was not significantly changed in cases with different tumor differentiation (Fig. S1). However, SIRT3 was gradually decreased from well- to poor-differentiated HCC (Fig. 4A). Percentage of cases with high SIRT3 expression was 24.4% in poor-differentiated HCC, noticeably lower than 43.5% in well-differentiated HCC (Fig. 4B).

### Correlation of SIRT3 Expression with Survival of Postoperative HCC Patients

**Figure 5:**
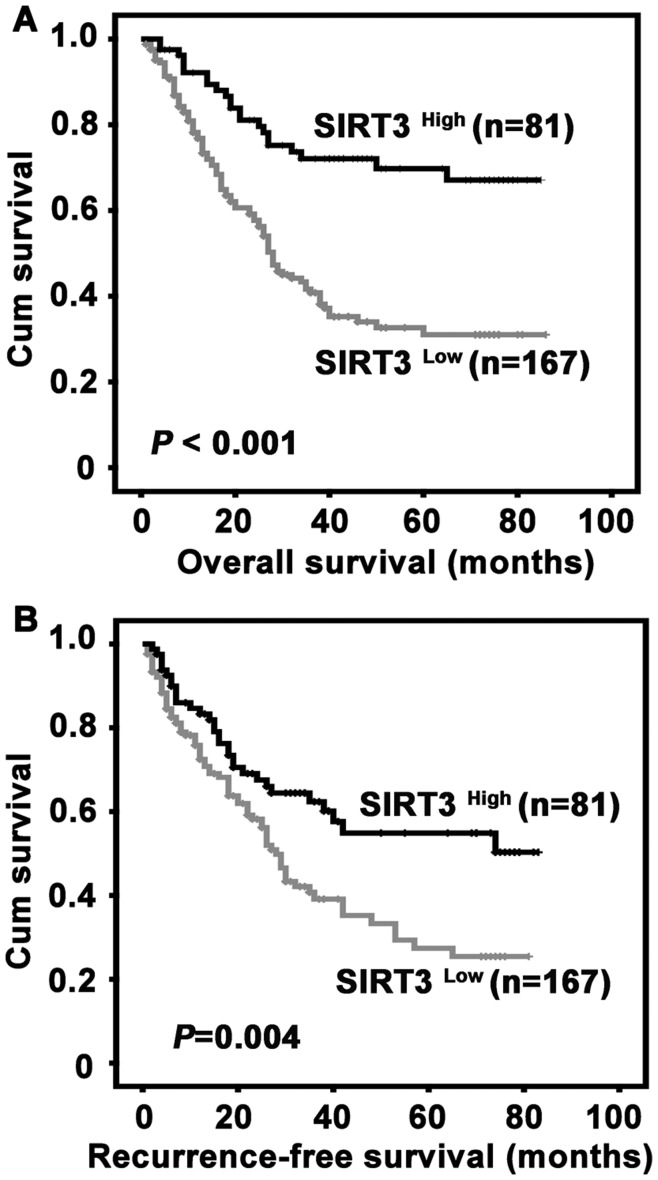
Correlation of low SIRT3 expression in HCC tissue with unfavorable overall survival and recurrence-free survival. Probabilities of overall survival **(A)** and recurrence-free survival **(B)** of total 248 HCC patients were analyzed by Kaplan-Meier survival analysis (log-rank test).

To determine whether SIRT3 expression was related to survival of HCC patients after surgical resection, Kaplan-Meier survival analyses were performed. Survival data were available for 248 patients. The average survival time was 40.9 months for the patients with low SIRT3 expression, while it was 65.0 months for patients expressed high level of SIRT3. Patients with low SIRT3 expression were likely to be with significantly shorter overall survival (*P*<0.01) (Fig. 5A) and recurrence-free survival (*P* = 0.004) (Fig. 5B).

**Figure 6:**
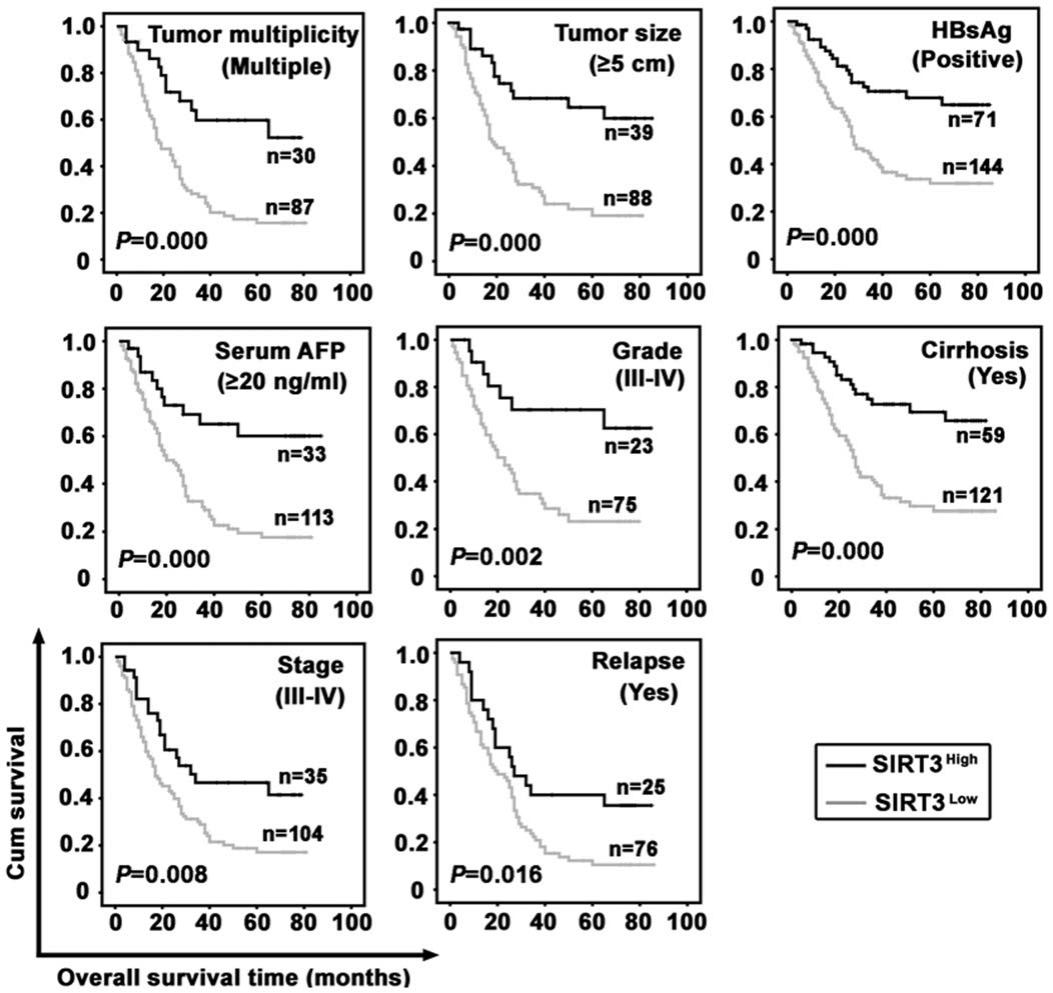
Relation of SIRT3 expression with overall survival in morphologic and pathological HCC subgroups. Survival analysis was performed in subgroups according to the factors that are attributed to worse outcome of HCC patients, using Kaplan-Meier survival analysis (log-rank test).

The impact of SIRT3 on prognosis was further evident in HCC patients subclassified by the factors attributed to worse outcome. The 8 subgroups of HCC patients were identified as ‘tumor multiplicity (multiple)’, ‘tumor size (≥5 cm)’, ‘HBsAg (positive)’, ‘serum AFP (≥20 ng/ml)’, ‘grade (III–IV)’, ‘cirrhosis (yes)’, ‘stage (III–IV)’, and ‘relapse (yes)’. Results revealed that patients with low SIRT3 expression in all subgroups survived shorter than those with high SIRT3 expression (Fig. 6). Unfortunately, it seemed that SIRT3 expression was not of significance in predicting the recurrence-free survival for these subgroups of HCC cases (Fig. S2). Our further study indicated that low SIRT3 expression in HCC patients with tumor size (<5 cm), grade (I–II), or stage (I–II) associated with poorer overall survival but not recurrence-free survival (Fig. S3).

### Univariate and Multivariate Analyses of Prognostic Variables in HCC Patients

To evaluate the representative of our samples, univariate analyses were applied. Results indicated that SIRT3, as well as tumor size, serum AFP level, tumor multiplicity, clinical stage, vascular invasion, tumor differentiation, and relapse, was responsible for outcome of HCC patient who underwent surgical resection (Tables 2 and S1).

**Table 2:** Univariate analysis of SIRT3 expression and clinicopathologic variables in 248 patients with primary hepatocellular carcinoma (log-rank test).

Variable	All cases	Overall survival (months)			Recurrence-free survival (months)		
		Mean	Median	*P* value	Mean	Median	*P* value
Age (years)^a^				0.963			0.997
<47.8	130	49.0	38.0		42.8	29.0	
≥47.8	118	48.8	40.0		43.8	38.0	
Gender				0.359			0.641
Female	28	52.5	NR		34.6	35.0	
Male	220	48.5	38.0		44.6	42.0	
HBsAg				0.722			0.298
Positive	215	49.5	38.0		43.1	32.0	
Negative	33	44.8	38.0		47.9	42.0	
AFP (ng/ml)				**0.000**			**0.000**
<20	102	65.8	NR		52.9	57.0	
≥20	146	37.7	27.0		36.1	25.0	
Cirrhosis				0.500			0.529
Yes	180	48.2	36.0		42.1	30.0	
No	68	51.7	NR		47.6	42.0	
Tumor size (cm)				**0.000**			**0.000**
<5	121	59.1	NR		53.4	74.0	
≥5	127	40.8	27.0		35.3	22.0	
Tumor multiplicity				**0.000**			**0.008**
Single	131	62.6	NR		49.8	42.0	
Multiple	117	35.3	24.0		36.7	26.0	
Differentiation				**0.003**			**0.024**
Well-Moderate	150	54.6	NR		47.7	42.0	
Poor- Undifferentiation	98	41.0	26.0		36.4	26.0	
Stage				**0.000**			**0.000**
I–II	109	70.9	NR		61.7	NR	
III–IV	139	33.4	21.0		31.0	22.0	
Vascular invasion				**0.000**			**0.000**
Yes	73	26.8	18.0		18.5	15.0	
No	175	58.2	NR		57.9	NR	
Relapse				**0.000**			
Yes	101	31.1	24.0				
No	147	64.2	NR				
SIRT3				**0.000**			**0.004**
Low expression	167	40.9	28.0		37.4	28.0	
High expression	81	65.0	NR		53.5	NR	

a Mean age;

NR, not reached; HbsAg, hepatitis B surface antigen; AFP, alpha-fetoprotein.

Multiple Cox regression analysis was conducted to determine the independent prognostic value of SIRT3. After adjusted for the prognostic factors established in univariate analysis, a significant correlation of low SIRT3 expression with worse overall survival (Hazard Ratio (HR) 0.555, *P* = 0.016) was observed (Table 3). However, SIRT3 was revealed not to be an independent marker of recurrence-free survival (Hazard Ratio (HR) 0.712, *P* = 0.123) for HCC patients (Table S2). Taken together, our data suggested SIRT3 as an independent prognostic biomarker for overall survival in postsurgical patients with primary HCC.

**Table 3:** Cox multivariate analyses of prognostic factors on overall survival.

Variable	β	SE	Hazard ratio (95%CI)	*P* value
Tumor multiplicity	0.141	0.266	1.152 (0.683–1.942)	0.596
Tumor size	0.116	0.213	1.124 (0.740–1.706)	0.585
AFP	0.750	0.229	2.117 (1.352–3.316)	**0.001**
Differentiation	−0.020	0.197	0.981 (0.667–1.442)	0.921
Vascular invasion	0.519	0.219	1.680 (1.093–2.582)	**0.018**
Stage	0.954	0.344	2.596 (1.322–5.100)	**0.006**
Relapse	0.807	0.219	2.241 (1.459–3.443)	**0.000**
SIRT3	−0.588	0.245	0.555 (0.344–0.897)	**0.016**

β, Regression coefficient; SE, standard error; CI, confidence interval; AFP, alpha-fetoprotein.

## Discussion

HCC is a heterogeneous cancer with a very high mortality. Searching for valuable biomarkers for HCC diagnosis and prognostic prediction has been attracting more and more interests. In recent years, many potential biomarkers have been disclosed to be of diagnostic value in HCC, such as serum DKK1 [24], serum anti-Ku86 [25], and microRNA panel [26]. On the other hand, studies revealed that altered expression of certain protein or mircoRNA could predict prognosis in HCC, such as ZBP-89 [20], FoxC1 [27], IGF-1 [28], and miR-139 [29]. Besides of proteins and microRNAs, other signatures of cancer, including DNA methylation [30], [31] and circulating tumor cell [32], have been also investigated to reveal their clinical significances in HCC diagnosis and prognosis. In the current study, we investigated the relevance and prognostic value of SIRT3 expression in a large cohort of primary HCC patients who underwent curative surgery.

Accumulating literatures support sirtuin family exerts essential functions in processes related to metabolism, such as aging and carcinogenesis [9], [33]. Out of seven members of sirtuin family, SIRT3 has been drawing particular attentions with regard to its impacts on mitochondrial function. To date, data suggest SIRT3 exhibits dichotomous functions dependent on cell contexts: either as tumor promoter or as tumor suppressor [34]. On one hand, SIRT3 plays a role of tumor promoter. SIRT3 prevented bladder cancer cells from growth arrest and senescence by targeting p53 to inhibit its activity [35]. SIRT3 abrogated stress-mediated apoptosis by deacetylating Ku70 which resulted in enhancement of Ku70-Bax interaction and prevention of Bax translocation to mitochondria [36]. Furthermore, downregulation of SIRT3 arrested OSCC cell proliferation and sensitized cancer cells to radiation and chemotherapy treatments [18]. On the other hand, SIRT3 functions as a tumor repressor. It has been reported that SIRT3 was required for JNK2-regulated apoptosis induced by selective silencing of Bcl-2 in HCT116 cells [37]. SIRT3 decreased ROS and maintained genomic stability to act as a tumor suppressor [38], [39]. Furthermore, MEFs with Sirt3^−/−^ were easily immortalized by infection with a single oncogene, and developed into subcutaneous xenograft tumor in nude mice once expressing *Myc* or *Ras* [17]. Moreover, SIRT3 deficiency in over one-year old mice resulted in development of estrogen- and progesterone-positive mammary tumors [17]. More recently, SIRT3 was shown to downreguated MDM2 to prevent p53 degradation, which subsequently inhibited HCC cell growth [19]. In our study, SIRT3 was dramatically decreased in HCC cell lines and more than 200 HCC tissue samples, at both mRNA and protein levels. Further data demonstrated that poorly-differentiated tumors expressed less SIRT3 than well-differentiated tumors in most of HCC cases. Moreover, low SIRT3 expression was positively significantly correlated to advanced clinical stage, high serum AFP, multiple tumor numbers and higher relapse rate. Collectively, these data indicated loss of SIRT3 was coincident with tumor progression, which suggests SIRT3 as a tumor suppressor in HCC. However, the mechanistic nature of SIRT3 in inhibiting HCC progression remains poorly unknown, and it therefore deserts a challenge for future investigation.

Low SIRT3 expression has been identified as a poor independent prognostic factor for both overall survival and recurrence-free survival in postsurgical HCC patients in this study. There is no previous study reporting the association between SIRT3 expression and prognosis in cancer. However, high SIRT1 expression was reported to connect to poor survival in diffuse large B-cell lymphoma [40], gastric carcinoma [41], and breast cancer [42]. Furthermore, downregulation of SIRT1 in HCC resulted in abrogation of cell proliferation and enhanced sensitivity to doxorubicin treatment by induction of senescence or apoptosis [43], [44]. The finding that patients with high SIRT3 expression survived longer could be supported by that SIRT3 was capable of inducing apoptosis. In colorectal carcinoma, SIRT3 was response to stress-induced apoptosis [37]. In leukemia cells, increasing SIRT3 contributed to apoptosis caused by Kaempferol treatment [45]. In HCC cells, overexpression of SIRT3 led to activation of JNK and the resulting apoptosis [19].

Importantly, low SIRT3 expression associated to markedly shorter period of clinical recurrence. This observation suggests that more attention should be paid to HCC patients with low SIRT3 expression during and after the process of therapy, though further investigation and validation are required before a clinical practice.

Interestingly, the prognostic effect of SIRT3 is especially strong in stratified survival analysis of HCC, according to the factors attributed to worse outcome. Low SIRT3 expression therefore could identify a subgroup of HCC patients who accompany with worse prognosis. Strikingly, low SIRT3 expression could also predict poor overall survival of HCC patients with tumor size (<5 cm), grade (I-II), or stage (I-II). This suggested that decrease of SIRT3 in HCC could be of clinical significance for predicting outcome of surgical treatment in a subset of HCC patients.

In summary, our study provided vigorous evidence that low SIRT3 expression was frequently present in HCC, particularly in those poor-differentiated cases. Decrease of SIRT3 in HCC was significantly correlated with clinical stage, serum AFP level, tumor differentiation and tumor multiplicity, indicating that SIRT3 might be involved in HCC progression. Importantly, although little information of SIRT3 in hepatocarcinogenesis is available, our study suggests that low expression of SIRT3, as detected by IHC, may be useful for predicting the postoperative survival of HCC patients.

## Supporting Information

Figure S1 **Percentages of high SIRT3 expressions in noncancerous tissue adjacent to HCC tissue were indicated by histogram.**
(TIF)

Figure S2 **Relation of SIRT3 expression with recurrence-free survival in pathological HCC subgroups.** Survival analysis was performed in subgroups according to the factors that are attributed to worse outcome of HCC patients, using Kaplan-Meier survival analysis (log-rank test).
(TIF)

Figure S3 **Survival analysis of SIRT3 expression in HCC patients with serum AFP (<20 ng/ml), or tumor size (<5 cm), or stage (I–II), or grade (I–II).**
(DOC)

Table S1 **Hazard ratios of univariate analysis.**
(DOC)

Table S2 **Cox multivariate analyses of prognostic factors on recurrence-free survival.**
(DOC)
