# Supplementary figures and images for: Low SIRT3 Expression Correlates with Poor Differentiation and Unfavorable Prognosis in Primary Hepatocellular Carcinoma
Source: PLoS One. 2012 Dec 14;7(12):e51703. doi: 10.1371/journal.pone.0051703 (PMC3522714; doi:10.1371/journal.pone.0051703)

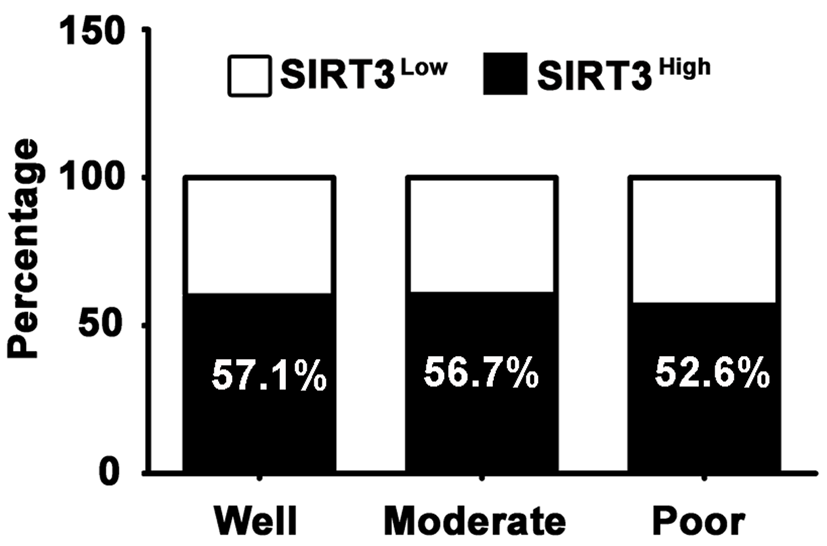

Supplement: Figure S1 — Percentages of high SIRT3 expressions in noncancerous tissue adjacent to HCC tissue were indicated by histogram. (TIF) [file pone.0051703.s001.tif]

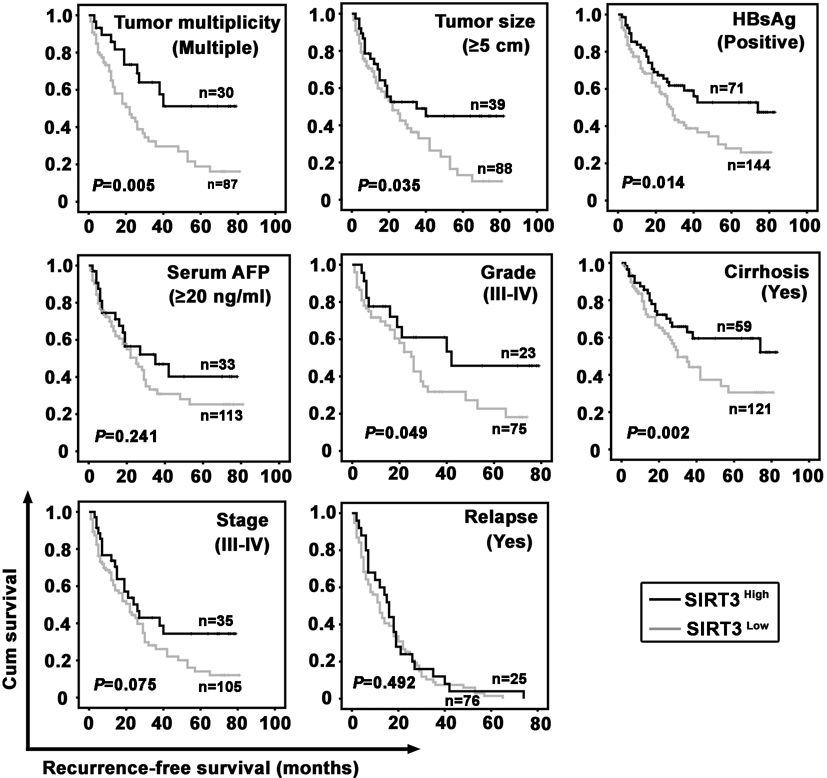

Supplement: Figure S2 — Relation of SIRT3 expression with recurrence-free survival in pathological HCC subgroups. Survival analysis was performed in subgroups according to the factors that are attributed to worse outcome of HCC patients, using Kaplan-Meier survival analysis (log-rank test). (TIF) [file pone.0051703.s002.tif]
