# Supplementary material for: Low SIRT3 Expression Correlates with Poor Differentiation and Unfavorable Prognosis in Primary Hepatocellular Carcinoma
Source: PLoS One. 2012 Dec 14;7(12):e51703. doi: 10.1371/journal.pone.0051703 (PMC3522714; doi:10.1371/journal.pone.0051703)
Supplement: Figure S3 — Survival analysis of SIRT3 expression in HCC patients with serum AFP (<20 ng/ml), or tumor size (<5 cm), or stage (I–II), or grade (I–II). (DOC) [file pone.0051703.s003.doc]

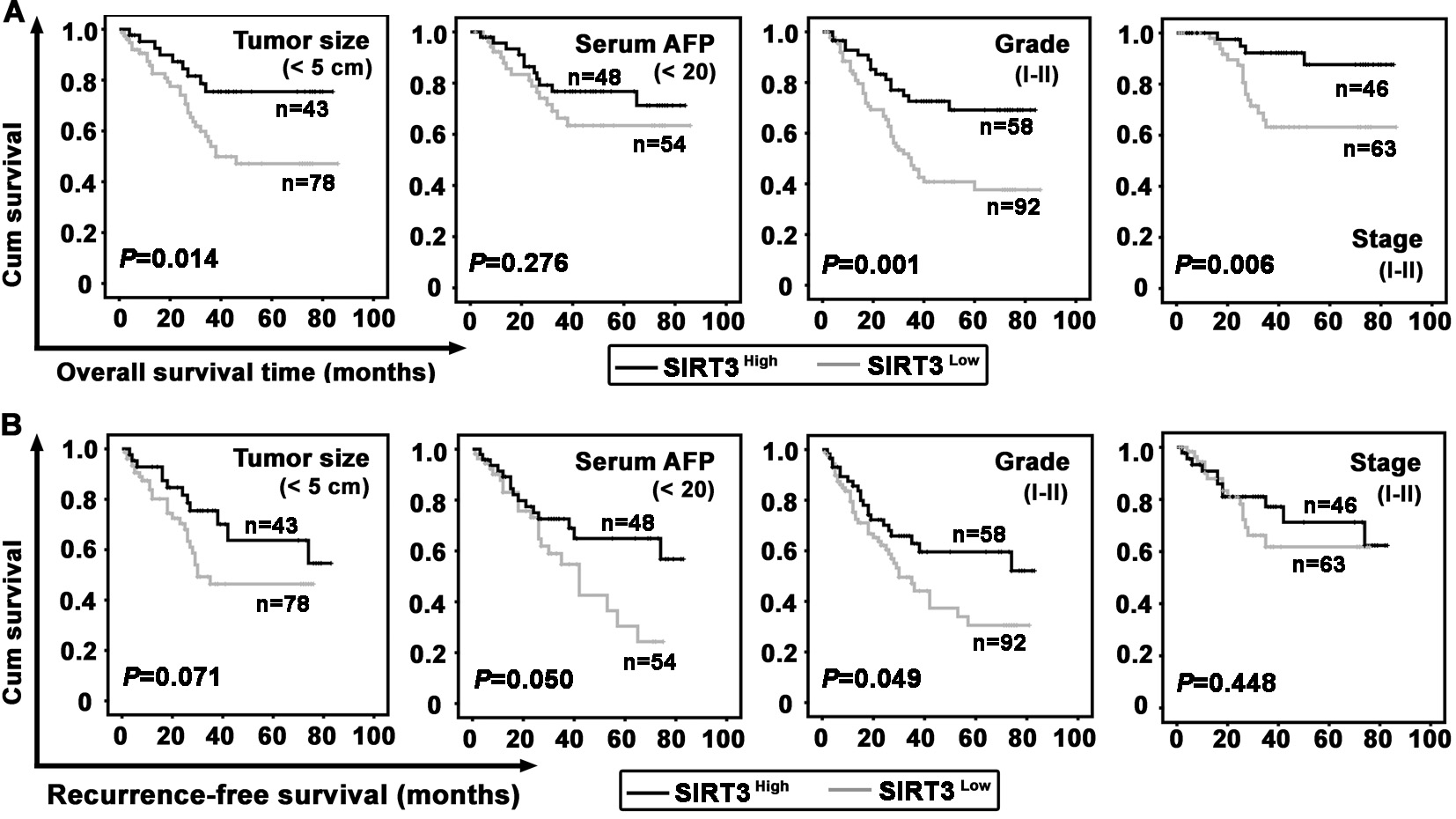


**Figure S3. Survival analysis of SIRT3 expression in HCC patients with serum AFP (< 20 ng/ml), or tumor size (< 5 cm), or stage (I-II), or grade (I-II).**
