# Supplementary material for: Low SIRT3 Expression Correlates with Poor Differentiation and Unfavorable Prognosis in Primary Hepatocellular Carcinoma
Source: PLoS One. 2012 Dec 14;7(12):e51703. doi: 10.1371/journal.pone.0051703 (PMC3522714; doi:10.1371/journal.pone.0051703)
Supplement: Table S1 — Hazard ratios of univariate analysis. (DOC) [file pone.0051703.s004.doc]

**Table S1.** Hazard ratios of univariate analysis.

| **Variable** | **Hazard ratio (95%CI)** | |
| --- | --- | --- |
|  | overall survival | recurrence-free survival |
| Age | 0.750 (0.430-1.397) | 1.024 (0.712-1.471) |
| Gender | 1.061 (0.712-1.451) | 1.141 (0.651-2.000) |
| HBsAg | 0.907 (0.527-1.561) | 1.385 (0.743-2.581) |
| AFP | 3.229 (2.096-4.973) | 2.009 (1.355-2.978) |
| Cirrhosis | 1.154 (0.758-1.758) | 1.144 (0.749-1.747) |
| Tumor size | 2.078 (1.416-3.050) | 2.056 (1.396-3.029) |
| Tumor multiplicity | 2.883 (1.949-4.265) | 1.646 (1.133-2.391) |
| Differentiation | 1.710 (1.187-2.463) | 1.526 (1.049-2.218) |
| Stage | 5.317 (3.280-8.620) | 3.604 (2.320-5.599) |
| Vascular invasion | 3.204 (2.211-4.643) | 4.942 (3.365-7.258) |
| Relapse | 4.148 (2.786-6.175) | 17.608 (10.096-30.710) |
| SIRT3 | 0.343 (0.215-0.546) | 0.644 (0.430-0.966) |

CI, confidence interval; HbsAg, hepatitis B surface antigen; AFP, alpha-fetoprotein.
