# Supplementary material for: Low SIRT3 Expression Correlates with Poor Differentiation and Unfavorable Prognosis in Primary Hepatocellular Carcinoma
Source: PLoS One. 2012 Dec 14;7(12):e51703. doi: 10.1371/journal.pone.0051703 (PMC3522714; doi:10.1371/journal.pone.0051703)
Supplement: Table S2 — Cox multivariate analyses of prognostic factors on recurrence-free survival. (DOC) [file pone.0051703.s005.doc]

**Table S2.** Cox multivariate analyses of prognostic factors on recurrence-free survival.

| **Variable** | **β** | **SE** | **Hazard ratio (95%CI)** | ***P* value** |
| --- | --- | --- | --- | --- |
| Tumor multiplicity | 0.075 | 0.237 | 1.078 (0.677-1.716) | 0.751 |
| Tumor size | 0.345 | 0.215 | 1.411 (0.927-2.149) | 0.108 |
| AFP | 0.436 | 0.208 | 1.546 (1.029-2.324) | **0.036** |
| Differentiation | 0.115 | 0.197 | 1.122 (0.763-1.652) | 0.558 |
| Vascular invasion | 1.299 | 0.235 | 3.665 (2.313-5.806) | **0.000** |
| Stage | 0.425 | 0.327 | 1.529 (0.806-2.900) | 0.194 |
| SIRT3 | -0.339 | 0.220 | 0.712 (0.463-1.096) | 0.123 |

β, Regression coefficient; SE, standard error; CI, confidence interval; AFP, alpha-fetoprotein.
